# Optimal dose of vigorous physical activity on cardiorespiratory and perceptual response for sedentary youths using internal load monitoring

**DOI:** 10.3389/fphys.2024.1406402

**Published:** 2024-09-20

**Authors:** Haohan Yu, Yue Gao, Jiaxin Liang, Yiming Fan, Shan Jiang

**Affiliations:** ^1^ Division of Sports Science and Physical Education, Tsinghua University, Beijing, China; ^2^ School of Leisure Sports and Tourism, Beijing Sport University, Beijing, China; ^3^ Physical Education Department, Kunming University of Science and Technology Oxbridge College, Kunming, Yunnan, China; ^4^ College of P.E and Sports, Beijing Normal University, Beijing, China; ^5^ Department of Sports Science and Physical Education, The Chinese University of Hong Kong, Shatin, Hong Kong SAR, China

**Keywords:** physical activity, sedentary behavior, cardiorespiratory fitness, psychological responses, training load

## Abstract

**Introduction:**

Vigorous physical activity (VPA) has been demonstrated to enhance cardiorespiratory fitness (CRF) in sedentary college students more effectively than other PA. However, differences in training volume may affect this outcome. This study examines the physiological, psychological, and internal training load (ITL) characteristics of VPA with varying volumes in a single session.

**Methods:**

Thirty sedentary college students were divided into three groups: high-intensity interval training (HIIT), sprint interval training (SIT), and threshold training (THR). PA process was monitored. The study measured various cardiorespiratory parameters, including heart rate (HR), respiratory waveform and amplitude, respiratory rate (RR), tidal volume (TV), minute ventilation volume (VE), fractional concentration of oxygen in end-tidal gas (O2%), fractional concentration of end-tidal carbon dioxide (CO2%), global oxygen consumption (VO2), carbon dioxide discharge (VCO2), and the amount of carbon dioxide in the air. The following physiological indicators were measured: carbon dioxide discharge (VCO2), Oxygen pulse (OP), and respiratory exchange ratio (RER). Additionally, subjective perception indicators were recorded, including the feeling scale (FS), rating of perceived exertion (RPE), and dual-mode model (DMM). The session-RPE (s-RPE) and Edward's TRIMP were used to measure ITL.

**Results:**

There were no significant differences in HR across the three conditions. THR had the highest level of TV (*p* = 0.043), but RR was significantly lower than that of HIIT and SIT (*p* < 0.01). HIIT had the highest levels of VO2, VCO2, O2%, and OP (*p* < 0.05). RPE was higher in HIIT and SIT compared to THR (*p* < 0.01), but the difference in FS was not significant. The DMM time-domain trajectories were similar in HIIT and THR. The correlation between exercise intensity, RPE, and FS was highest in THR group (r = 0.453, r = −0.58, r = −0.885). ITL did not show a significant difference between three conditions, but TRIMP and s-RPE readings were opposite in magnitude.

**Conclusion:**

This study proposes that using an appropriate amount of THR to foster interest and adaptive strength during the PA habit establishment period, incorporating HIIT to enhance exercise efficiency during the adaptation period, and implementing SIT to reduce the monotony may effectively enhance the cardiorespiratory fitness of sedentary college students and establish PA habit.

## Introduction

Sedentary behavior (SB) has become a common daily habit worldwide due to the development of modern society. SB is defined as any waking behavior that involves energy expenditure of ≤1.5 METs while sitting or reclining (World Health Organization, 2020). According to the World Health Organization’s (WHO) Global Status Report on Physical Activity 2022, 28% of the world’s population engages in SB. This behavior has become prevalent among college students, particularly on university campuses, due to academic pressures. SB is a common habit among college students (World Health Organization, 2022). Numerous studies have shown that SB not only reduces interest in exercise but also causes physical symptoms such as poor circulation, disruption of endocrine balance, and musculoskeletal abnormalities. These symptoms can lead to various diseases, including cardiovascular and metabolic syndromes (Curran et al., 2023; Pinto et al., 2023). The American Heart Association (AHA) has demonstrated that reduced cardiorespiratory fitness (CRF) is the primary risk factor for SB. This negatively impacts the physical functioning and quality of life of college students (Lavie et al., 2019).

In response to the above, the WHO guidelines on physical activity and sedentary behavior indicate that regular physical activity (PA), as a non-pharmacological tool, can effectively increase stroke volume, maximal oxygen consumption, and oxygen pulsation, thereby improving oxygen uptake, vascular wall elasticity, and cardiac output, and promoting the development of CRF (World Health Organization, 2020). PA, as a non-pharmacological tool, can effectively increase stroke volume, maximal oxygen consumption, and oxygen pulsation, thereby improving oxygen uptake, vascular wall elasticity, and cardiac output, and promoting the development of CRF. However, as the scientific level of exercise increases, a large number of studies have found that the effects of different types of exercise on CRF vary. Among these, vigorous physical activity (VPA) has been shown to provide individuals with greater cardiorespiratory stimulation in a single session, making its long-term benefits superior to those of other exercises. For instance, Rachelle discovered that VPA not only improved cardiorespiratory fitness more effectively than moderate-intensity continuous training (MICT), but also saved time during exercise (Sultana et al., 2019). In a year-long study, Taylor discovered that individuals who participated in VPA experienced greater improvements in VO2max and exercise tolerance compared to those who engaged in MICT (Taylor et al., 2020). Similarly, Wisløff found that VPA had a more significant effect on brachial artery flow and mitochondrial function, leading to a higher level of CRF improvement from a mechanistic perspective (Wisløff et al., 2007). Therefore, due to the characteristics of ‘high benefit and short time’, various VPA programs have gained attention in the field of sports training and clinical research.

While the beneficial impact of VPA on CRF has been extensively proven, the advancement of technology and quantification of training loads has led to numerous studies monitoring the subjective and physiological loads of individuals during exercise through internal training load (ITL) (McLaren et al., 2018). This makes it more challenging to enhance the resolution and precision of our training. It has been discovered that different training modes, even at similar intensities, can still result in differences in physiological and psychological parameters among individuals (Ekkekakis et al., 2002; Mooren et al., 2023). Therefore, setting the training volume is a crucial factor in developing training programs and prescribing exercises. VPA can be divided into three types of training based on differences in training capacity: high-intensity interval training (HIIT), sprint interval training (SIT), and threshold training (THR). Research indicates that each of the three types of exercise has distinct physiological and psychological effects on individuals. Michael’s study found that the HIIT group had a 4% higher time-trial performance than the SIT group, and that maximal aerobic power and velocity were significantly improved in the HIIT group (Rosenblat et al., 2020). Alejandro demonstrated that SIT improved individuals’ strength more than HIIT did (Pérez-Castilla et al., 2023), while Tsung found that HIIT and THR had similar effects on CRF in sedentary college students (Chiang et al., 2023). Up to now, while the variations in athletic performance enhancement, strength quality improvement, and physical health interventions among the three groups have been preliminarily confirmed, there is still a lack of monitoring reports and characterization of CRF parameters, subjective perception, and ITL during exercise.

To better quantify the dosage characteristics and physiological mechanisms of cardiorespiratory responses, exercise perception, and subjective/physiological loads of sedentary college students due to differences in training capacity in VPA, this study was conducted to monitor the process of three exercise conditions. This study aims to determine the optimal dose of VPA for improving CRF, explain the physiological mechanisms behind it, and provide a scientific exercise prescription and theoretical basis for promoting cardiopulmonary health, cultivating interest in exercise, and enhancing exercise efficiency in sedentary college students. Based on the rationale provided above, this study’s hypotheses are: (I) The expression levels of HR may be similar in all three conditions; (II) THR exhibits the highest oxygen uptake while SIT exhibits the lowest; (III) Subjective fatigue levels are THR > HIIT > SIT, while the opposite is true for the perception of movement; (IV) THR exhibits the highest subjective and physiological loads, whereas HIIT and SIT do not differ significantly; and (V) The subjective and physiological loads are the highest in THR, whereas HIIT and SIT do not differ significantly.

## Materials and methods

This study was a randomized controlled trial conducted at the Exercise Human Science Laboratory of Beijing Normal University. As this study is an exercise physiology monitoring experiment, the purpose is to assess the expression characteristics of physiological and psychological indexes of sedentary adolescents during different modes of VPA, and the outcome indexes are all continuous variables and have no absolute categorical variables, so there is no blank control group. The aim was to identify the most effective VPA program for improving CRF and perceptions of exercise experience in sedentary adolescents. During the experiment, participants were randomized into HIIT, SIT, and THR groups. The experimental environment was set up in a relatively constant temperature laboratory (23°C–26°C) with supervised power cycling exercises. The experimental period was from February 2023 to April 2023. Tests are standardized between 13:00 and 17:00. In order to familiarize the participants with the exercise protocol and the equipment, and to reduce the probability of risky events during the experiment, all personnel were asked to perform at least 1 pre-experiment and exercise practice. Prior to the experiment, participants were asked to maintain a normal routine and refrain from strenuous exercise for 24 h prior to the exercise session, and to refrain from consuming large amounts of food and water for 2 h prior to the experiment to ensure optimal hydration.

### Participants

The present study was based on the WHO guidelines on physical activity and sedentary behavior for sedentary populations (World Health Organization, 2020), and was conducted according to the Bassett protocol using the International Physical Activity Questionnaire (IPAQ). The International Physical Activity Questionnaire (IPAQ) initially recruited 35 participants who self-reported more than 8 h of sedentary time per day and less than 30min of moderate to vigorous physical activity (Bassett, 2003). All participants were screened to ensure that they had no prior long-term occupational training or exercise habits. Twenty-seven male and eight female adolescents between the ages of 20 and 25 were included. However, due to the large difference in numbers between genders and the age range of the eight females, the numbers did not fit a normal distribution. Therefore, in this study, in order to reduce systematic error and heterogeneity to improve the reliability of the results, only 27 male adolescents whose age fit the normal distribution were included in the final experiment.

The allocation of subjects to the different exercise groups was performed in a blinded manner using a randomized lottery. Prior to the start of the experiment, all subjects were asked to sign an informed consent form, which included an acknowledgement of the experimental risks, no history of psychiatric disorders, cardiovascular disease, muscle contraindications, or adverse drug experiences. At the same time, participants were informed that they would be required to undergo at least two or more exercise interventions, including the pre-experiment and the formal experiment. In addition, all experimenters were blinded to ensure the privacy of the participants. The experiment was approved by the Human Experimentation Ethics Committee of the School of Physical Education and Sport, Beijing Normal University (approval number: TY20220629, see Supplement one for details). The experiment was conducted in strict accordance with the requirements and procedures of the Institutional Review Board, and the demographic information is shown in Table 1.

**TABLE 1:** Participants’ physical characteristics.

	HIIT	SIT	THR
Numbers (n)	11	7	9
Age (yr)	22.82 ± 1.47	23.57 ± 1.62	23 ± 1
Body mass (kg)	77 ± 7.39	76.14 ± 5.93	76 ± 5.92
Height (m)	1.77 ± 0.05	1.77 ± 0.04	1.78 ± 0.05
BMI (kg/m2)	24.43 ± 1.42	24.41 ± 1.42	23.86 ± 1.46
HRrest (bpm)	76.27 ± 4.69	77.29 ± 5.94	77.33 ± 5.45
HRmax (bpm)	191.18 ± 1.47	190.43 ± 1.62	189.56 ± 1.13
Sedentary behavior (hour/day)	8.63 ± 1.28	8.71 ± 1.12	8.25 ± 0.86
MVPA (min/day)	26.18 ± 6.19	27.42 ± 6.24	26.77 ± 5.85

### Procedures

The exercise interventions consisted of power cycling. The subjects’ RPE, FS, and FAS scores were recorded every minute until completion. During the implementation, supervisors strictly followed the target HR intervals specified by the AHA to control exercise intensity. Failure to achieve the target intensity will result in an invalid experimental procedure.

#### Baseline testing

Before the experiment, the subjects’ height and weight were measured to calculate their BMI (kg/m2). The maximum exercise capacity of each individual was determined through graded exercise tests (GXT) performed on a cycle ergometer (Chengdu Taimeng Technology Co., Ltd.). The participants were warmed up with a resistance of 50 W for 2 min, followed by an increase of 20 W every minute until complete exhaustion (cycle speed below 50 rpm). Verbal encouragement was provided by the experimenter to ensure the integrity of the experiment. VO2 and HR were recorded during exercise using the same brand of human physiological metabolic analyzer and POLAR Verity Sense, with a sampling frequency of one time/s. The highest values of VO2max and HRmax were recorded within 15–30 s after the end of the test, provided that the individual’s RERmax was greater than 1.10 or real-time HR was less than or equal to 220 minus the individual’s age (Olney et al., 2018). These values were then used to determine the intensity of subsequent exercise sessions.

#### High-intensity interval training

The HIIT group used Wisløff’s classic protocol with modifications (Wisløff et al., 2007). The whole program lasted about 20 min Including a 5 min warm-up, during which the heart rate was kept above 50% HRmax. This was followed by five sets of interval training. Each set consisted of a 2min high-intensity sprint at 85% HRmax or higher. Bridging to 3min of active recovery exercise, participants reduced the intensity of the active recovery activity on their own but did not stop exercising.

#### Sprint interval training

The SIT group used Kimberly’s protocol exclusively (Wood et al., 2016). The duration of the entire program was approximately 20 min Which included a 5 min warm-up during which the heart rate was maintained above 50% HRmax. Unlike HIIT, the SIT group reduced the duration of sprinting and recovery and bridged 10 sets of interval training. Each set consisted of a 30-s high-intensity sprint at an intensity above 85% HRmax. This was interspersed with 1.5 min of active recovery exercise, where participants reduced the intensity on their own for active relaxation activities but did not stop exercising.

#### Threshold training

In the THR group, the exercise mode was traditional high-intensity target heart rate training, so the Chiang classic program was used (Chiang et al., 2023). The duration of the whole process was 20 min Compared to HIIT and SIT, the whole process of THR did not have any recovery phase. First, participants performed a 5-min warm-up to elevate HR to within the target intensity range of 70%–80% HRmax (≈95% VT1). Afterwards, continuous exercise without intervals was performed until the end of training.

### Measurements

#### Heart rate monitoring

The POLAR Verity Sense was used as HR testing instrument in this study. It was worn on the left arm throughout the exercise and had a sampling frequency of one time/s. During the wearing process, the biosensing contact surface of the POLAR watch was uniformly cleaned and maintained to avoid any interference with HR data collection. The watch was worn on the top of the left side of the arm in a strictly regulated position to prevent any special reasons, such as body fluid obstruction, from affecting the results. To ensure data quality, the experimental staff will conduct a 3–5 min data collection test before the official experiment using the POLAR PC software. This will allow them to check the real-time HR collection status and make any necessary adjustments to the equipment and experiment steps based on technical issues that may arise on site.

#### Respiratory metabolism measurement

The HPS-10 human metabolism monitor (Chengdu Taimeng Technology Co., Ltd.) was used to monitor the respiratory index during the exercise. The subjects wore wireless respiratory and metabolic waveform monitoring masks throughout the exercise process. The normal body movements of the subjects were not affected during the exercise. The respiratory parameters included in this study were respiratory waveform and amplitude, respiratory rate (RR), tidal volume (TV), minute ventilation volume (VE), fractional concentration of oxygen in end-tidal gas (O2), the volume of oxygen in end-tidal gas (O2), fractional concentration of end-tidal carbon dioxide (CO2), global oxygen consumption (VO2), carbon dioxide discharge (VCO2), oxygen pulse (OP), and respiratory exchange ratio (RER). The experimenter manually recorded the cardiopulmonary parameters every minute and entered them into the experiment log.

#### Perceptual response assessment

Hardy & Rejeski’s feeling scale (FS) (Hardy and Rejeski, 1989) and Borg’s rating of perceived exertion scale-10 items (RPE) (Li et al., 2023) were used to evaluate subjective perception and provide an overall assessment of subjects’ subjective motor sensations during a single training session. Additionally, a circular model-based dual-mode model (DMM) was employed to illustrate the dose-response relationship between RPE and FS in two orthogonal/bipolar dimensions (Ekkekakis et al., 2002). In the case of the RPE and FS included in this study, the four quadrants of the DMM represent four psychological states: the vitality-pleasure quadrant (quadrant 4), the fatigue-pleasure quadrant (quadrant 1), the vitality-dullness quadrant (quadrant 3), and the fatigue-dullness quadrant (quadrant 4). Outside of these quadrants, there are no significant mental fluctuations. By observing the time-domain trajectory of the DMM, experimenters can clearly understand the changes in the two-factor dynamic relationship between exerciser experience to fatigue during the exercise process, and provide data clues for increasing interest in exercise and adjusting the exercise prescription. During the experiment, athletes reported their scores at 1-min intervals and at the peak of each motor state. The experimenter recorded the scores. After the experiment, the data were organized, analyzed, and used to establish DMM and calculate ITL.

#### Internal training load evaluation

In this study, the subjective and physiological loads of each group of subjects were calculated by training impulse. In this case, training impulse used Foster’s session RPE (s-RPE) (2001) (Foster, 1998) based on subjective fatigue level with Edwards’ five-zone Edwards’ TRIMP (SHRZ) (1993) (Castagna et al., 2011) based on HR characteristics. Unlike the traditional Banister’s TRIMP (1991) (Mekjavic et al., 1991), the SHRZ divides the HR intervals and thus not only calculates an individual’s load per unit time in a more refined manner, but also reflects the load distribution characteristics during exercise.

①The formula for session-RPE is as follows:

session−RPE=T×RPE

②The formula for Edward’s TRIMP is as follows:

$$TRIMP=\left(T\times 1\right)+\left(T\times 2\right)+\left(T\times 3\right)+\left(T\times 4\right)+\left(T\times 5\right)$$

**Table:** 

HR zones (%HRmax)	Coefficient
90%–100%	5
80%–90%	4
70%–80%	3
60%–70%	2
50%–60%	1

T represents the duration of the exercise. TRIMP, values are calculated by multiplying the duration within each interval by the corresponding weighting factor. The sum of the TRIMP, values for each interval is the total TRIMP, value for the training period. The RPE, is calculated using Borg’s CR-10 RPE.

### Statistical analysis

All data were counted and analyzed using IBM SPSS Statistics_26 and descriptive statistics and reliability tests were performed after normality tests. All individual results were then combined and presented as mean ± standard deviation. First, the overall mean differences of all outcome variables were compared and calibrated by one-way analysis of variance (ANOVA). The mean values of HR, TV, RR, and VE represent the total cardiorespiratory load imposed by the three VPAs per unit of time, while the mean values of O2%, CO2%, VO2, VCO2, RER, and VCO2/HR reflect the respiratory metabolism and oxygen uptake of an individual during a single bout of exercise and are used to evaluate the effects of acute VPAs on CRF in a single session. RPE and FS reflect the effects of different exercise regimens on an individual’s subjective mood and experience, while TRIMP and s-RPE represent the level of perceptual and physiological load experienced by an individual during exercise and are used to evaluate the effects of a single training session. Second, repeated-measures one-way ANOVA and multivariate analysis of variance (MANOVA) were used to compare and analyze the expression levels of each index at different time points to explore the time-domain changes of individual cardiorespiratory response, perceptual load, and physiological load during different VPA and to provide a reference basis for improving the training program. HR, FS, and RPE were analyzed by Pearson’s coefficients to investigate the effects of training volume on subjective mood of exercisers at similar exercise intensities (HRmax). Subjective mood of exercisers at similar exercise intensities (HRmax). The analysis of effect sizes was assessed with the Greenhouse-Geisser Assumed Sphericity test, and the results of cases that did not meet the hypotheses were corrected using the Greenhouse-Geisser method. Post hoc tests for each outcome were performed using the Bonferroni test, with a significance threshold of *p* < 0.05.

## Results

There were no risk events in this study. Compliance was 100% in the HIIT group, three withdrew during the SIT for 70% compliance, and one was unable to complete the test during the THR and voluntarily requested to be transferred to the HIIT group for training for 90% compliance. The results of the cardiorespiratory fitness test indicated that HR and ventilatory efficiency were similar across all three exercise conditions. However, there were notable differences in gas exchange rates, particularly in VO2, VCO2, and OP, which increased with the training volume but did not exceed the expected levels when the exercise type remained constant. The results of the study showed that intermittent exercise elicited higher levels of fatigue compared to continuous exercise. However, the participants’ perceptions of exercise were generally low and did not significantly differ among the three groups. The training load results indicate that although the subjective and physiological loads of the three conditions were not significantly different, the magnitude of the readings was opposite to each other, and the load distribution characteristics were closely related to the training volume.

### Heart rate characteristics during different training

**FIGURE 1:**
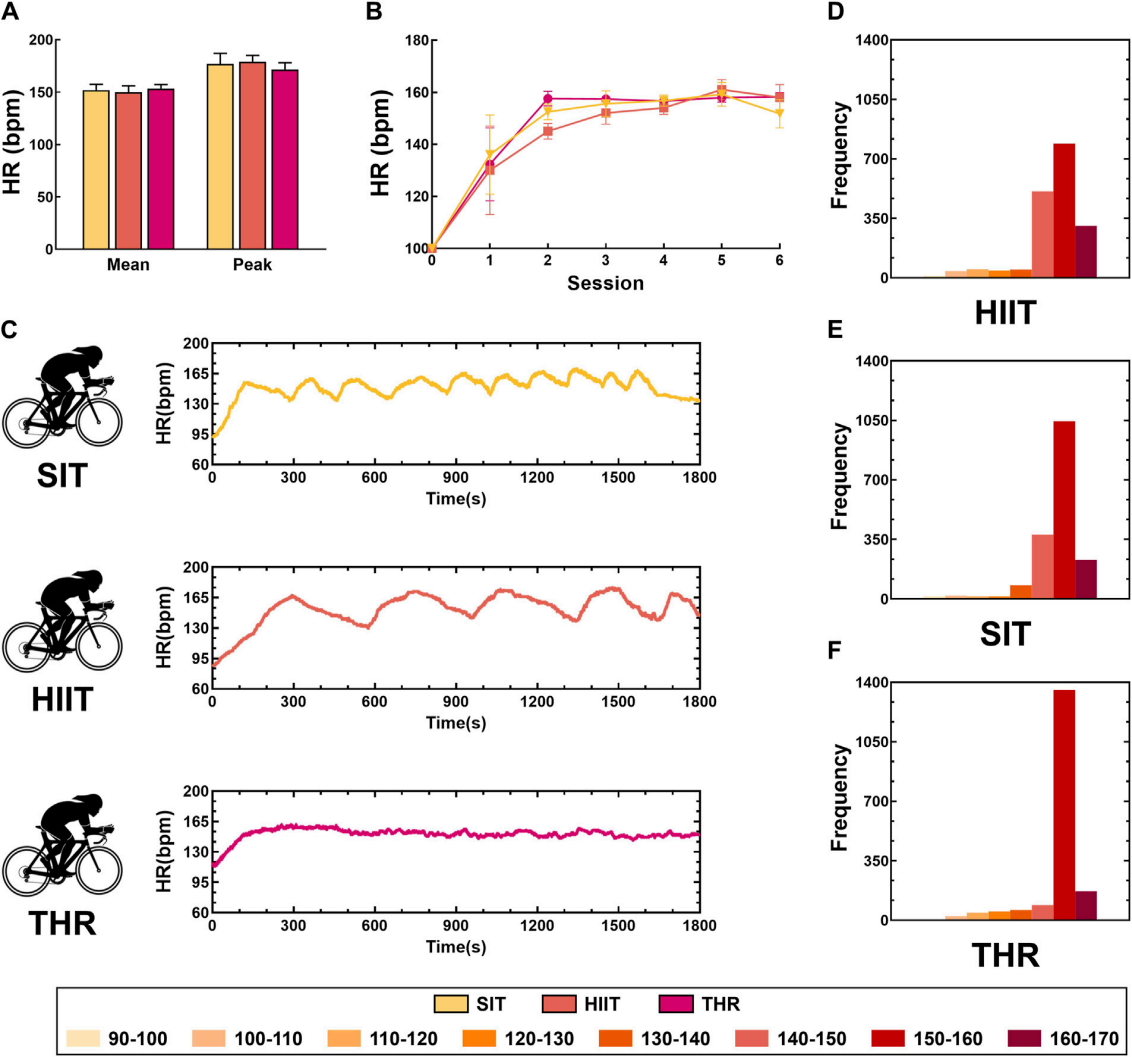
Characteristics of heart rate distribution intervals and time domain changes in different states. Note: **(A)** Expression of HRmean and HRpeak in different states; **(B)** Time-domain characteristics of HR divided by session; **(C)** Time-domain characteristics of HR in different states; **(D)** HR intervals in HIIT; **(E)** HR intervals in SIT; **(F)** HR intervals in THR.

The results showed that the HR time domain and amplitude characteristics of the three exercise conditions differed significantly (Figure 1A). Among them, SIT had the smallest amplitude and shortest peak period, HIIT had moderate amplitude and larger period than SIT, and THR was relatively stable without large fluctuations. ANOVA *post hoc* analyses showed that (Figure 1B), even though the HRpeak of THR was lower than that of HIIT and SIT, there was no statistically significant difference among the three conditions (*p* > 0.05). MANOVA repeated measures showed (Figure 1C) that the HRs of the three conditions were not all equal (HIIT: F = 28.915; SIT: F = 10.965; THR: F = 28.509). In the HIIT group, HR increased significantly in sessions 1–2 (*p* < 0.01), was more moderate in sessions 2–4 (*p* > 0.08), but climbed again in sessions 4–5 (*p* = 0.01), and then did not fluctuate significantly (*p* = 0.165); in the SIT group, HR increased significantly in the pre-exercise period (sessions 1–3) (*p* < 0.02), and did not change significantly in the late period (sessions 4–6) (*p* > 0.05); the THR group only increased in the early period (sessions 1–2), and did not change significantly in the late period (sessions 4–6) (*p* > 0.05); and in the THR group, it was only in the early period (sessions 1–2), and did not change significantly. (*p* < 0.02) in the SIT group in the pre-exercise period (session 1–3), but no significant change in the later period (session 4–6) (*p* > 0.05); HR in the THR group climbed only briefly in the early period of exercise (session 1–2) (*p* < 0.01), and then stabilized thereafter (*p* > 0.1). The HR interval characteristics show (Figure 1D) that the HRs of the three conditions are mainly distributed in the range of 140–170 bpm. Although the intensity of the three conditions is similar, the HR interval characteristics are different. Among them, 150-160bpm accounted for the case of HIIT < SIT < THR, while 140-150bpm and 160-170bpm accounted for the opposite of the former. All the details are shown in Table 2.

**TABLE 2:** Expression status of heart rate in different states/stages and distribution intervals.

HIIT								
	Mean	Peak	S-1	S-2	S-3	S-4	S-5	S-6
HR (bpm)	149.93 ± 6.44	178.55 ± 5.73	129.77 ± 13.16	145.16 ± 9.36	152.29 ± 10.85	154.13 ± 6.66	160.55 ± 6.07	157.75 ± 5.16
	HR-Range							
	90–100	100–110	110–120	120–130	130–140	140–150	150–160	160–170
Frequency (s)	11	40	50	43	49	509	790	306
Proportion (%)	0.61	2.22	2.78	2.39	2.73	28.31	43.94	17.02

SIT								
	Mean	Peak	S-1	S-2	S-3	S-4	S-5	S-6
HR (bpm)	151.95 ± 5.4	176.86 ± 10.12	136.04 ± 10.54	152.46 ± 5.64	155.57 ± 7.75	156.74 ± 2.46	159.17 ± 6.16	151.72 ± 12.11
	HR-Range							
	90–100	100–110	110–120	120–130	130–140	140–150	150–160	160–170
Frequency (s)	15	20	15	15	81	377	1045	230
Proportion (%)	0.83	1.11	0.83	0.83	4.51	20.97	58.12	12.79

THR								
	Mean	Peak	S-1	S-2	S-3	S-4	S-5	S-6
HR (bpm)	153.32 ± 3.73	171.56 ± 6.46	132.34 ± 10.11	157.56 ± 2.68	157.39 ± 5.11	156.57 ± 4.65	157.93 ± 5.57	158.17 ± 7.93
	HR-Range							
	90–100	100–110	110–120	120–130	130–140	140–150	150–160	160–170
Frequency (s)	0	25	45	52	60	90	1355	171
Proportion (%)	0	1.39	2.5	2.89	3.34	5.01	75.36	9.51

### Respiratory metabolism characteristics during different training

**FIGURE 2:**
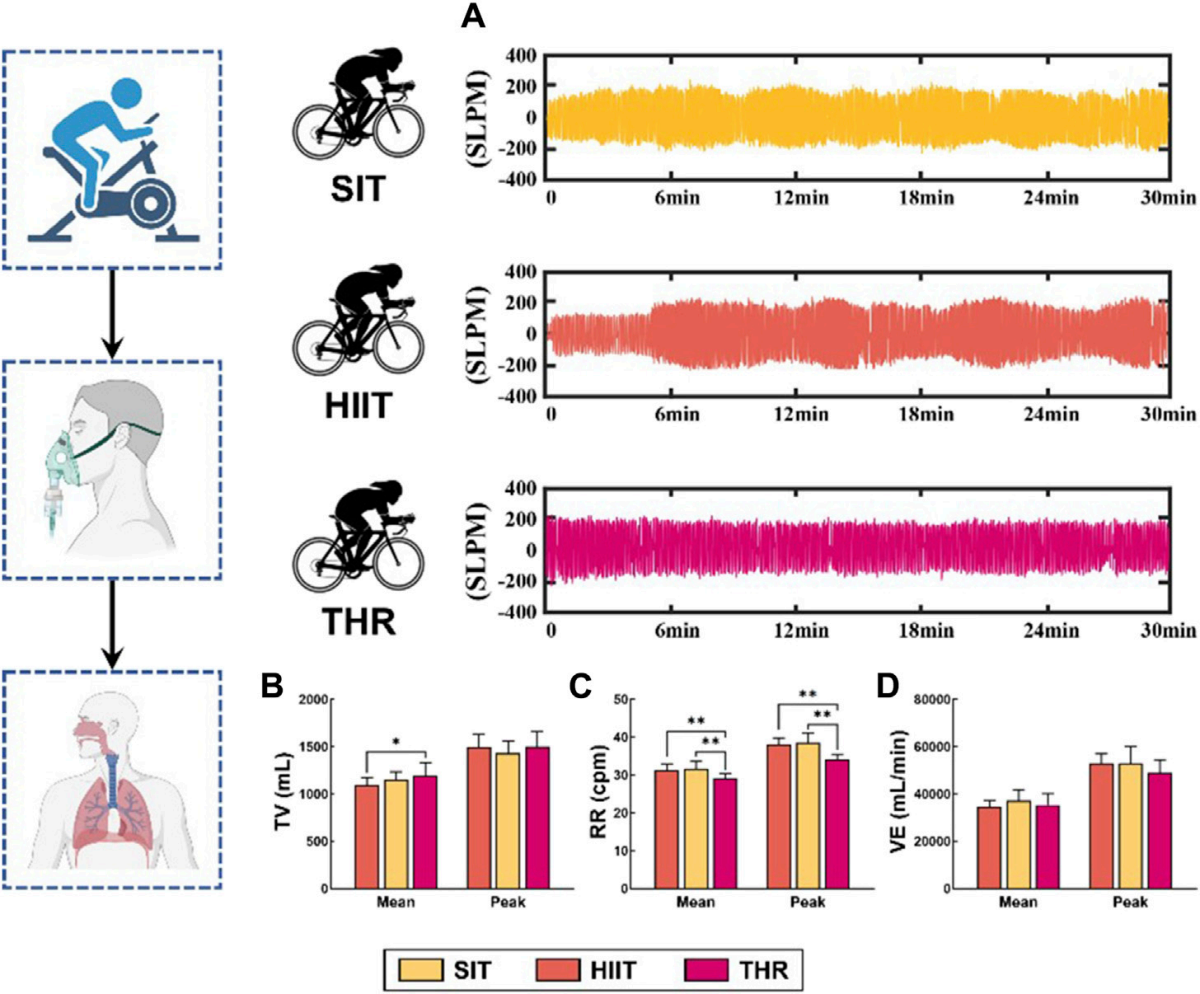
Respiratory metabolism in different states. Note: **(A)** Characteristics of respiratory flow rate in different states; **(B–D)**: Expression of respiratory rate/tidal volume/ventilation per minute in different states.

**FIGURE 3:**
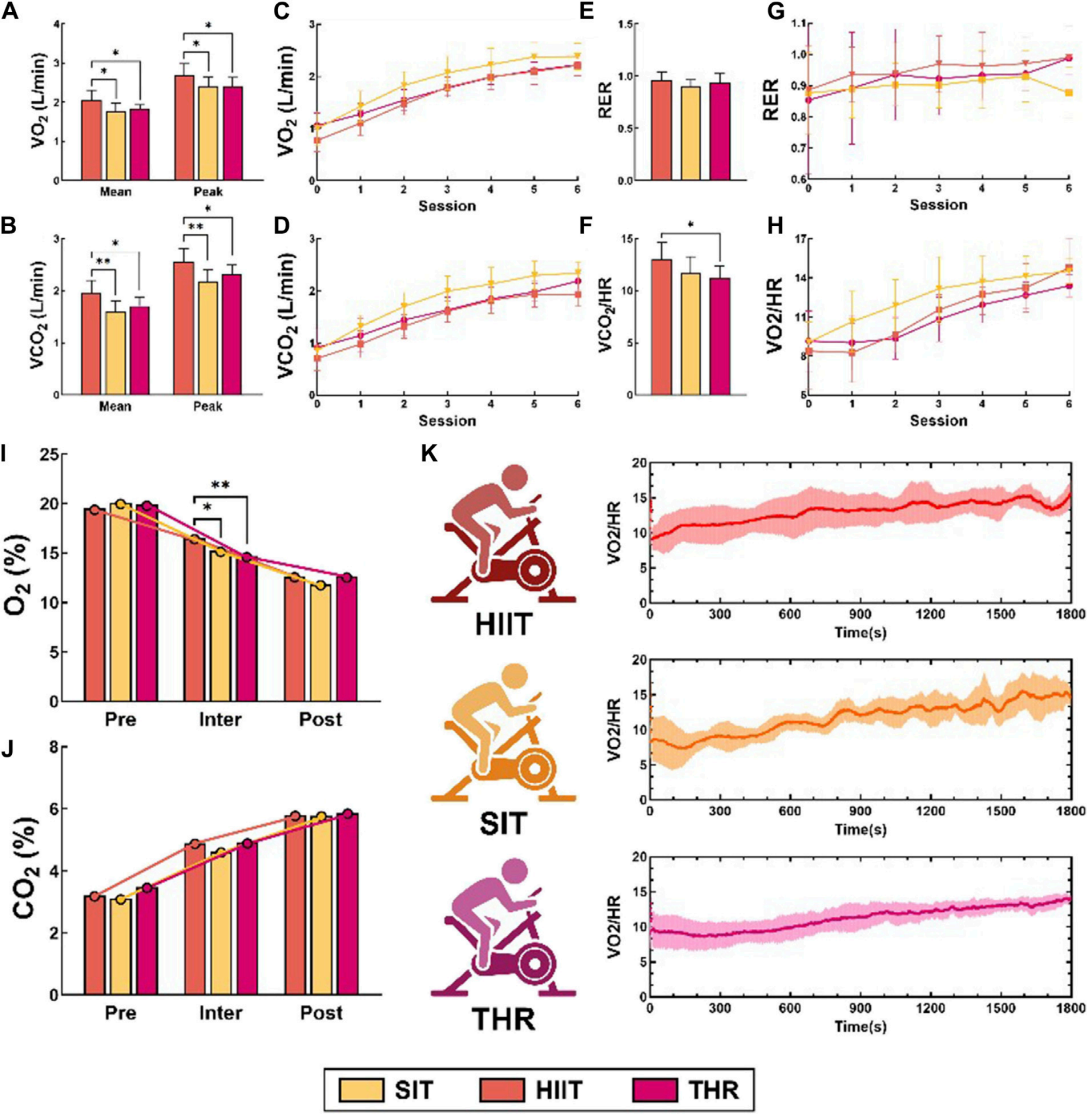
Gas exchange in different states. Note: **(A–H)** Mean and peak oxygen uptake in different states; **(B)** Mean and peak carbon dioxide output in different states; **(C)** Time-domain characteristics of oxygen uptake in different states; **(D)** HR intervals in HIIT; **(E)** HR intervals in SIT; **(F)** HR intervals in THR. oxygen uptake in different states; **(D)** Time-domain characteristics of carbon dioxide output in different states; **(E)** Respiratory exchange ratio in different states; **(F)** Oxygen pulse in different states; **(G)** Time-domain characteristics of respiratory exchange ratio; **(H)** Time-domain characteristics of oxygen pulse; **(I)** Expression post-exercise; **(K)** Oxygen pulse in different states.

Respiratory metabolic characteristics showed (Figure 2A) that the amplitude of respiratory waveforms in the three exercise conditions was similar to that of HR, characterized as: THR was the most stable; SIT had a small amplitude and a short period; and HIIT had a large amplitude and a long period. The results of the *post hoc* analysis of the ANOVA showed that, although the TVmean of the three conditions, VEmean/peak were not statistically significant, but the TVmean of THR group was higher than that of HIIT group (*p* = 0.043) (Figures 2B–D), and the difference of RR was significant (*p* < 0.01), which was as follows: SIT > HIIT>THR (Figure 2C). Gas exchange status showed (Figures 3A, B, I, J) that the mean and peak expression levels of VO2, VCO2 and O2% were significantly higher in HIIT than in the other two exercises (VO2mean: HIIT-SIT: *p* = 0.010, HIIT-THR: *p* = 0.018; VO2peak: HIIT-SIT: *p* = 0.038, HIIT-THR: *p* = 0.018; VO2peak: HIIT-SIT: *p* = 0.038, HIIT-THR: *p* = 0.018). 0.038, HIIT-THR: *p* = 0.020; VCO2mean: HIIT-SIT: *p* = 0.002, HIIT-THR: *p* = 0.012; VCO2peak: HIIT-SIT: *p* = 0.001, HIIT-THR: *p* = 0.023; O2%: HIIT-SIT: *p* = 0.027, HIIT-THR: *p* = 0.001), but SIT and THR were not statistically significant.

**FIGURE 4:**
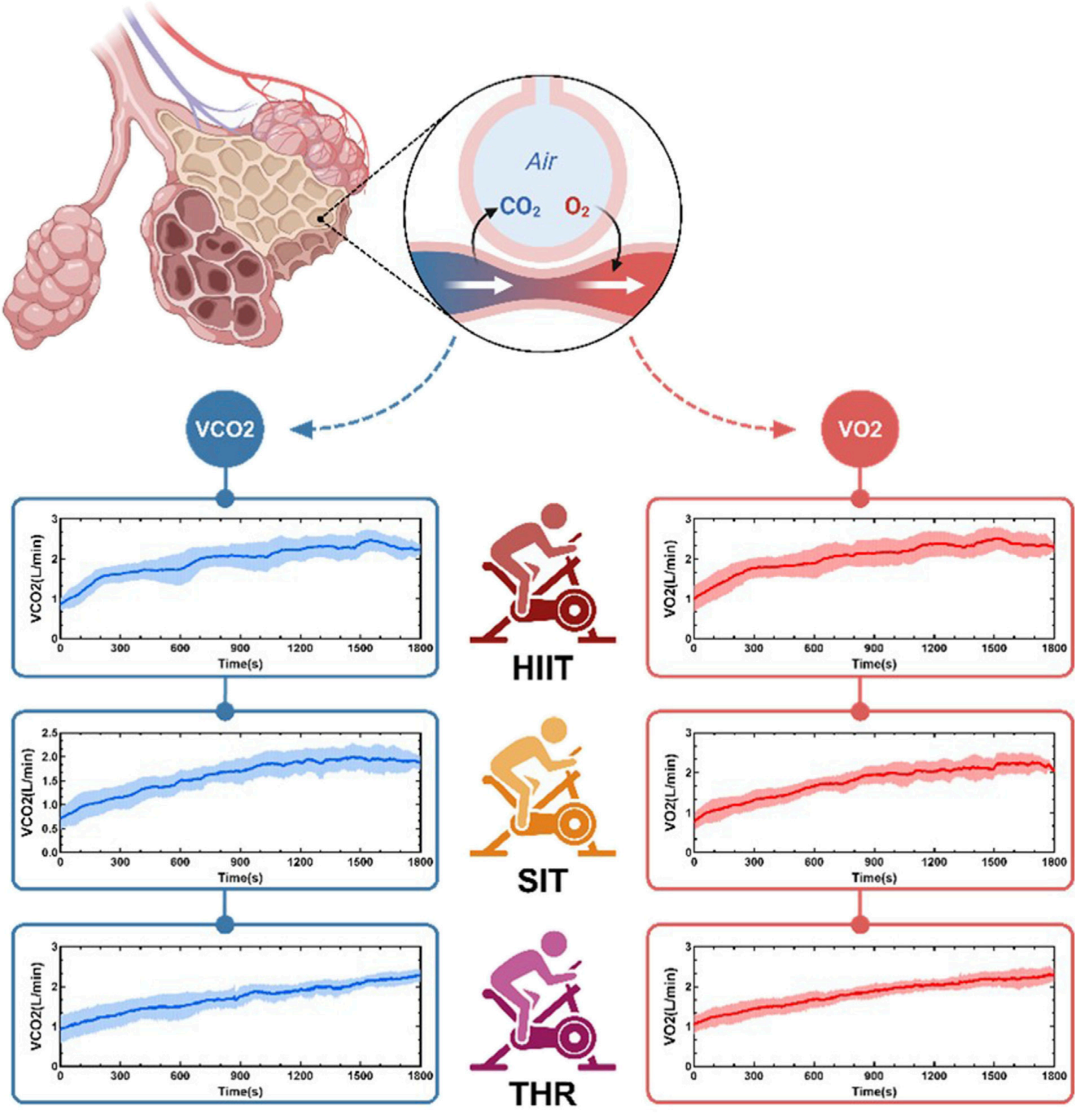
Time-domain characteristics of O2 and CO2 in different states of gas exchange.

MANOVA repeated measurements (Figures 3C, D, I, J) showed that VO2, VCO2, and CO2% increased consistently across all conditions, except for VCO2, which did not increase significantly at the end of the HIIT and SIT training sessions (sessions 5–6), while O2% decreased (VO2: HIIT: F = 72.401; SIT: F = 95.713; THR: F = 46.682, *p* < 0 05; VCO2: HIIT: F = 94.457, SIT: F = 94.457, THR: F = 46.682, *p* < 0 05). 72.401; SIT: F = 95.713; THR: F = 46.682, *p* < 0.05; VCO2: HIIT: F = 94.457, SIT: F = 73.434, THR: F = 41.470, *p* < 0.05; O2%: HIIT: F = 213.728, SIT: F = 96.630, THR: F = 157.413, *p* ≤ 0.01; CO2%: HIIT: F = 221.969, SIT: F = 97.721, THR: F = 117.618, *p* ≤ 0.01). The rest of the results showed that the RER was similar for THREE conditions (Figures 3E), with no significant fluctuations across the motions in the remaining phases (Figures 3G), except for a significant increase in THR at the end of the session (session 5–6) (F = 1.440, *p* = 0.011). Notably, OP in HIIT was significantly higher than THR (*p* = 0.011) (Figures 3F). In particular, HIIT increased significantly (F = 17.082, *p* < 0.05) in the pre-exercise period (session 1–3) and exceeded THR (*p* = 0.013) at session 2, while SIT and THR continued to increase (SIT: F = 44.490, *p* < 0.05; THR: F = 28.165, *p* < 0.05) (Figures 3H). The details of the time-domain variations of VO2 and VCO2 are shown in Figure 4. All the details are shown in Table 3.

**TABLE 3:** Respiratory metabolism levels in different states/stages.

HIIT										
	Mean	Peak		S-1	S-2		S-3	S-4	S-5	S-6
TV (mL)	1090.31 ± 78.28	1497.17 ± 137.09								
RR (cpm)	31.21 ± 1.72	38.09 ± 1.71								
VE (mL/min)	34,528.95 ± 2808.75	52,712.14 ± 4203.38								
VO2 (L/min)	2.05 ± 0.26	2.68 ± 0.29		1.42 ± 0.3	1.83 ± 0.27		2.07 ± 0.32	2.23 ± 0.31	2.38 ± 0.27	2.38 ± 0.25
VCO2 (L/min)	1.96 ± 0.24	2.56 ± 0.24		1.32 ± 0.21	1.7 ± 0.26		2 ± 0.29	2.13 ± 0.32	2.29 ± 0.28	2.34 ± 0.21
RER	0.96 ± 0.08			0.94 ± 0.09	0.94 ± 0.1		0.97 ± 0.09	0.96 ± 0.11	0.97 ± 0.09	0.99 ± 0.1
VO2/HR	13.01 ± 1.61			10.64 ± 2.34	11.88 ± 1.99		13.17 ± 2.45	13.69 ± 1.97	14.14 ± 1.55	14.52 ± 0.95
	Pre	Inter		Post						
O2% (%)	19.48% ± 1.07	16.52% ± 1.97		12.62% ± 1.42						
CO2% (%)	3.20% ± 0.35	4.89% ± 0.69		5.78% ± 0.27						

SIT										
	Mean	Peak		S-1		S-2	S-3	S-4	S-5	S-6
TV (mL)	1150.9 ± 84.33	1429.3 ± 128.67								
RR (cpm)	31.69 ± 1.99	38.54 ± 2.42								
VE (mL/min)	37,118.64 ± 4519.29	52,782.57 ± 7201.28								
VO2 (L/min)	1.78 ± 0.2	2.41 ± 0.23		1.1 ± 0.24		1.47 ± 0.2	1.79 ± 0.2	1.99 ± 0.24	2.09 ± 0.25	2.21 ± 0.19
VCO2 (L/min)	1.6 ± 0.21	2.17 ± 0.24		0.98 ± 0.26		1.32 ± 0.23	1.61 ± 0.2	1.82 ± 0.25	1.94 ± 0.25	1.93 ± 0.22
RER	0.9 ± 0.06			0.89 ± 0.09		0.9 ± 0.07	0.9 ± 0.07	0.92 ± 0.09	0.93 ± 0.08	0.88 ± 0.08
VO2/HR	11.7 ± 1.56			8.26 ± 2.27		9.65 ± 1.37	11.56 ± 1.17	12.75 ± 1.55	13.25 ± 1.87	14.77 ± 2.22
	Pre	Inter		Post						
O2% (%)	20.04% ± 0.84	15.23% ± 2.13		11.82% ± 1.32						
CO2% (%)	3.11% ± 0.63	4.61% ± 0.72		5.77% ± 0.35						

THR										
	Mean	Peak		S-1		S-2	S-3	S-4	S-5	S-6
TV (mL)	1189.52 ± 138.23	1499.32 ± 163.79								
RR (cpm)	29.06 ± 1.23	34.15 ± 1.33								
VE (mL/min)	35,349.3 ± 4831.29	49,064 ± 5340.27								
VO2 (L/min)	1.82 ± 0.11	2.39 ± 0.23		1.28 ± 0.22		1.54 ± 0.21	1.78 ± 0.15	1.98 ± 0.14	2.12 ± 0.16	2.22 ± 0.21
VCO2 (L/min)	1.71 ± 0.17	2.32 ± 0.17		1.15 ± 0.32		1.45 ± 0.3	1.64 ± 0.24	1.85 ± 0.19	1.98 ± 0.16	2.19 ± 0.15
RER	0.93 ± 0.09			0.89 ± 0.18		0.94 ± 0.15	0.92 ± 0.12	0.93 ± 0.08	0.94 ± 0.04	0.99 ± 0.05
VO2/HR	11.2 ± 1.18			9.02 ± 2.1		9.35 ± 1.59	10.82 ± 1.71	11.94 ± 1.38	12.68 ± 0.98	13.39 ± 0.83
	Pre	Inter		Post						
O2% (%)	19.89% ± .94	14.68% ± 1.53		12.63% ± 1.07						
CO2% (%)	3.47% ± 0.67	4.92% ± 0.65		5.86% ± 0.35						

### Characterization of motion perception and training load

**FIGURE 5:**
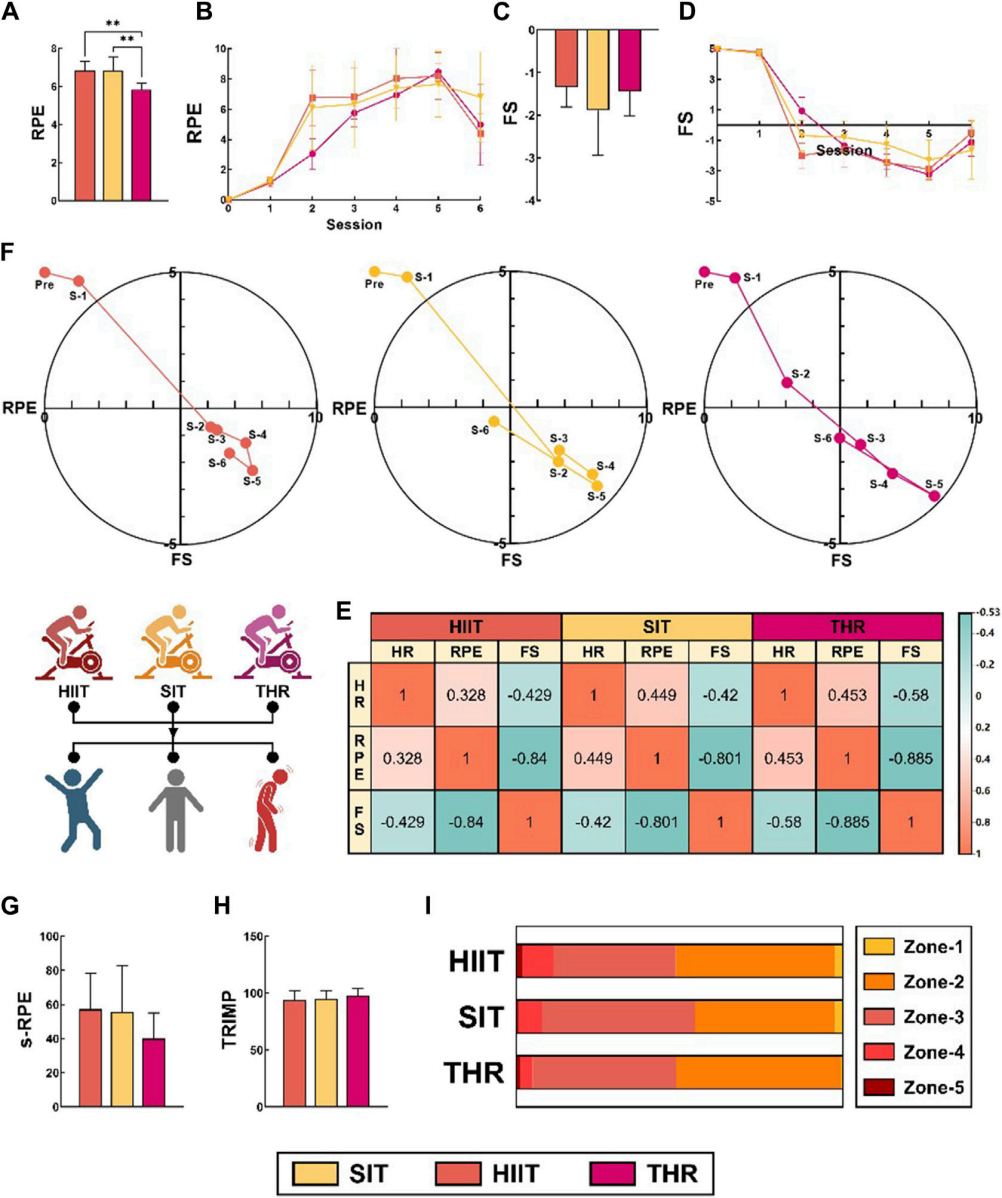
Exercise perception and training load condition in different states. Note: **(A–D)**: Expression levels and time domain characteristics of RPE and FS in different states; **(E)**: Correlations between exercise intensity, subjective fatigue, and exercise perception; **(F)**: DMM-based time-domain changes of subjective fatigue and exercise perception in different states; **(G, H)**: Expression levels of s-RPE with TRIMP in different states; **(I)**: Characteristics of the load distribution of different exercises in the intensity interval of Edward’s TRIMP.

The subjective perception results showed that individuals’ RPE in THR was significantly lower than HIIT (*p* < 0.01) and SIT (*p* = 0.001), while FS in three conditions was not significantly different (Figures 5A, C). MANOVA repeated measures showed (Figures 5B, D) that RPE in all three conditions showed a continuous increase (HIIT: F = 471.656, SIT: F = 166.956, SIT: F = 166.956, SIT: F = 166.956, SIT: F = 166.956) and a substantial decrease at the end of the exercise (session 5–6). Conditions showed a continuous increase in RPE and a significant decrease at the end of exercise (session 5–6) (HIIT: F = 471.656, SIT: F = 166.953, THR: F = 407.549). Both HIIT and SIT flattened in the middle of the exercise (session 2–3) and increased significantly (*p* < 0.05) in the early (session 1–2) and late (session 3–6) periods, while THR continued to increase (*p* < 0.01). FS, on the contrary, showed a continuous decrease and changed from decrease to increase at the end of the exercise (HIIT: F = 283.307, SIT: F = 102.914, THR: F = 305.824). The three trends remained similar, with HIIT and SIT decreasing slowly in the middle of the exercise period (session 2–3), then decreasing sharply in the early (session 1–2) and late (session 3–6) periods (*p* < 0.05), and THR decreasing throughout the exercise period (*p* < 0.01).

The DMM results show (Figures 5F) that the time-domain trajectories of the three conditions are similar, all of them start from the vigorous-pleasant quadrant, move to the fatigue-unpleasant quadrant at the lower right, and gradually approach the vigorous-unpleasant quadrant. Unlike THR, which remained in the vigorous-pleasant quadrant at the second sextile, SIT entered the vigorous-unpleasant quadrant at the end of the phase. Correlation results showed (Figures 5E) that exercise intensity (HR) was positively correlated with subjective fatigue level. Was positively correlated with the correlation rank of THR > SIT > HIIT (r = 0.453, r = 0.449, r = 0.328). Sense of enjoyment, on the other hand, was negatively correlated with both exercise intensity and subjective fatigue level, and the latter correlation was stronger, as shown by the correlation ratings of THR < HIIT < SIT (FS-HR: r = −0.58, r = −0.429, r = −0.42; FS-RPE: r = −0.885, r = −0.84, r = −0.801). This implies that an increase in training volume directly mediates an increase in fatigue levels during high-intensity exercise, and that the onset of fatigue is the primary factor causing a decrease in enjoyment.

The results of the training loads showed that although the differences between the subjective and physiological loads (s-RPE/TRIMP) for the three exercise conditions were not statistically significant, the readings showed the opposite results for s-RPE and TRIMP, with the s-RPE magnitude relationship being: HIIT > SIT>THR, and TRIMP being: HIIT < SIT < THR (Figures 5G, H). SIT < THR (Figures 5G, H). According to Edward’s TRIMP, the load distribution of the three conditions is similar: the medium load (Zone 2–3) accounts for more than 80% of the load. The difference is that high load zones (Zones 4–5) and low load zones (Zone 1) are the most prevalent in HIIT, followed by SIT, whereas THR spends most of its time in moderate load zones (Zones 2–3). This phenomenon also suggests that the larger the training volume, the smaller the load interval span, and the smaller the training volume, the greater the load fluctuation (Figures 5I). All the details are shown in Table 4.

**TABLE 4:** Subjective perception and training load condition in different states/phases.

HIIT								
	Mean	Peak	S-1	S-2	S-3	S-4	S-5	S-6
RPE	6.86 ± 0.46	10 ± 0	1.25 ± 0.24	6.11 ± 2.8	6.35 ± 2.86	7.4 ± 2.16	7.65 ± 2.18	6.8 ± 2.99
FS	−1.34 ± 0.46	−4.18 ± 0.75	4.69 ± 0.29	−0.69 ± 0.55	−0.8 ± 0.75	−1.27 ± 0.57	−2.29 ± 0.76	−1.65 ± 0.68

Training load								
	Mean			HR-Zone of SHRZ (%)				
				Zone-1	Zone-2	Zone-3	Zone-4	Zone-5
TRIMP	94.17 ± 8			1.75% ± 1.55	9.71% ± 4.5	37.44% ± 11.18	48.65% ± 13.27	2.45% ± 4.26
s-RPE	57.27 ± 21.02							

SIT								
	Mean	Peak	S-1	S-2	S-3	S-4	S-5	S-6
RPE	6.84 ± 0.7	10 ± 0	1.2 ± 0.31	6.77 ± 1.85	6.8 ± 1.96	8.03 ± 1.99	8.2 ± 1.55	4.4 ± 1.33
FS	−1.89 ± 1.06	−3.71 ± 0.95	4.8 ± 0.16	−2 ± 1.02	−1.57 ± 1.07	−2.46 ± 0.88	−2.89 ± 1.03	−0.51 ± 1.83

Training load								
	Mean			HR-Zone of SHRZ (%)				
				Zone-1	Zone-2	Zone-3	Zone-4	Zone-5
TRIMP	95.09 ± 7.19			0.7% ± 0.37	7.2% ± 4.53	46.83% ± 12.79	42.84% ± 12.23	2.42% ± 3.8
s-RPE	55.71 ± 26.99							

THR								
	Mean	Peak	S-1	S-2	S-3	S-4	S-5	S-6
RPE	5.84 ± 0.34	9.33 ± 0.71	1.13 ± 0.24	3.04 ± 1.01	5.76 ± 0.43	6.93 ± 0.26	8.47 ± 0.58	4.98 ± 2.7
FS	−1.44 ± 0.57	−3.89 ± 0.6	4.78 ± 0.25	0.93 ± 0.7	−1.36 ± 0.71	−2.42 ± 0.42	−3.24 ± 0.61	−1.11 ± 0.98

Training load								
	Mean			HR-Zone of SHRZ (%)				
				Zone-1	Zone-2	Zone-3	Zone-4	Zone-5
TRIMP	98.12 ± 5.91			1.19% ± 0.72	3.91% ± 1.68	44.04% ± 20.59	50.65% ± 20.64	0.21% ± 0.47
s-RPE	40 ± 15							

## Discussion

CRF is a crucial aspect of both physical and mental health. The AHA categorizes it as a ‘vital sign’ that determines an individual’s physiological function, exercise capacity, and quality of life (Ross et al., 2016). Decreased CRF is the primary risk factor for diseases caused by SB that can lead to all-cause-of-death (Lavie et al., 2019). While regular PA has been shown to improve CRF, differences between types of PA remain. Among college students, sedentary behavior is common due to limited exercise time caused by class schedules and academic pressures. To improve the efficiency of physical activity on their cardiorespiratory fitness and overall health, studies have shown the benefits of shortening exercise time and increasing exercise intensity (Winwood et al., 2024). However, this study concludes that simply increasing exercise intensity is not the most effective way to improve exercise efficiency. Therefore, this study compared three different forms of PA to VPA and explored the physiological and psychological significance of training volume in CRF and exercise experience. The study aims to provide more accurate exercise prescriptions and theoretical references for sedentary college students.

The initial discovery of this study is demonstrated through the CRF indicators, such as HR, VO2, and RER. These indicators represent an individual’s functional metabolic state and level of stimulation during exercise. Numerous studies have monitored their expression levels to assess the impact of various PAs on the enhancement of physiological and psychological health (Ji et al., 2024). The study results indicate that there were no significant differences in cardio metabolism between the HR and time domain characteristics of the three exercise conditions. All three conditions reached the ACSM recommended optimum cardiovascular training zone or above (Liguori, 2022). However, the THR group exhibited a more stable frequency of HR distribution over 20min compared to the other two groups. Therefore, hypothesis (I) can be accepted. During intermittent exercise (HIIT/SIT), individuals may be in a state with a large span of high and low intensities, making it difficult to effectively control HR within a fixed interval as in continuous exercise. Additionally, three conditions showed differences in respiratory metabolism. For instance, RR and OP were higher in intermittent exercise than in continuous exercise at THR. In the between-group comparison between HIIT and SIT, HIIT exhibited a higher level of respiratory metabolism (VO2/VCO2/O2%) than SIT, which contradicts hypothesis (II) and is consistent with previous findings. Stefano compared the effects of different volumes of intermittent exercise on cardiac vagal reactivity. The results showed that SIT and HIIT led to higher HRmean, lower HR recovery, and lower HR variability compared to THR. However, the differences between the groups were not statistically significant (Benítez-Flores et al., 2023). Fernando monitored cardiorespiratory responses to constant load exercise and interval exercise. The results showed that the HIIT group had higher levels of VE, VO2mean, and HRmean expression (Beltrami et al., 2021). Focusing on the training capacity aspect of interval exercise, Naves found that VO2peak and HRpeak were generally higher in individuals during HIIT and SIT than THR. However, HIIT had a significant enhancement of the right atrial reservoir and conduit functions, reducing radial and ventricular ejection fraction, and increasing the right ventricular ejection fraction during interval exercise. Additionally, HIIT resulted in a reduction of radial strain and pulmonary vascular resistance compared to SIT (Naves et al., 2019). Benjamin’s analysis found that in VPA, intervals shorter than 30 s may disrupt the respiratory pattern, leading to an inability of the individual’s cardiac output and oxygen transport efficiency to adapt to sudden increases in intensity (Wakefield and Glaister, 2009). Therefore, there is a correlation between the intensity and duration of intervals in PA and the individual CRF response. The study suggests that while interval exercise may provide a higher level of cardiorespiratory stimulation, training volume is a crucial factor in achieving this benefit. On the other hand, sedentary college students aim to improve physical activity efficiency by compressing training time. The study concludes that HIIT is the most effective way to achieve this goal.

The reason why HIIT can mediate higher levels of expression of CRF indicators in individuals than SIT with THR can be explained in terms of physiological basis. In our study, the mode of HIIT was set to 3 × 2, allowing subjects a longer period to adapt to the alternation between sprint and interval states. This led to adaptive changes in the myocardium, including thickening and structural adjustments of the ventricular wall, resulting in greater contractile force (Hanssen et al., 2011). At the same time, the process also increases the individual’s left ventricular end-diastolic volume (LVEDV) and left ventricular end-diastolic pressure (LVEDP) to a greater extent than the SIT. This allows for more blood to be accommodated during each diastolic period, thereby increasing (CO) to meet the body’s oxygen demand (Coates et al., 2023). Although there were no significant differences in HR among the three groups in this study, the SIT group had higher readings compared to the HIIT and THR groups. This is due to the sudden increase in sympathetic nervous system activity caused by the shorter and more intense stimulus in the SIT group. However, unlike HIIT, SIT does not allow enough time for the onset of sympathetic reactivation to mediate, resulting in insufficient myocardial adaptations. As a result, HR peaks and then rapidly declines after the sprint ends (O’Driscoll et al., 2018). Furthermore, the available evidence supports these findings. Huang compared the effects of single and periodic HIIT with THR on individual oxygen uptake levels by routine venous blood analysis and found that acute HIIT, compared with THR, increased erythrocyte osmotic deformability and aquaporin 1 (AQP1) levels more than THR, thereby mediating higher levels of oxygen transport efficiency and changes in myocardial adaptation. In contrast, after 6 weeks of intervention, HIIT attenuated the inhibitory effect of SB on erythrocyte membrane stability more than THR and increased stroke volume and umbilical artery flow velocity (Huang et al., 2019). Tsai also found that a single session of HIIT induced greater endothelium-dependent vasodilation and coronary perfusion in humans compared to SIT, while after 6 weeks of continuous intervention, participants in the HIIT group had higher densities of monocyte-derived endothelial progenitor cells, and hematopoietic stem cell densities were higher in participants in the HIIT group compared to SIT (Tsai et al., 2016). This also suggests that the training dose of HIIT induced an optimal cardiorespiratory response in individuals. On the other hand, mitochondrial function and thrombin regulation are also important mechanisms for improving CRF through HIIT. Chen found that HIIT can effectively improve neutrophil-derived microparticle (NDMP)-induced thrombin generation (TG) by down-regulating procoagulant factor expression during SB, thus reducing the risk of SB-induced thrombosis (Chen et al., 2015). Wu et al. demonstrated that increasing exercise intensity and adjusting training intervals can improve citrate synthase (CS) and succinate synthase. However, it is important to note that this may also increase the risk of SB-induced thrombosis. Wu et al. showed that increasing exercise intensity and adjusting training intervals can enhance the regulatory benefits of CS and succinate dehydrogenase (SDH) activities, as well as membrane potential (MP), which inhibits the elevation of matrix oxidant burden after SB. This suggests that HIIT improves mitochondrial bioenergetics and suppresses dynamic TG in platelets undergoing hypoxia more significantly than THR and SIT (Wu et al., 2017).

The study’s second finding is that varying training volumes in VPA result in differences in exercise experience. When developing exercise prescriptions for SB college students, it is crucial to consider both the physiological effects of exercise forms and the individual’s exercise experience. Research has demonstrated that having a positive experience is linked to an increased interest in exercise, while a negative experience can hinder the formation of exercise habits (Thøgersen-Ntoumani et al., 2023). In this study, it was observed that RPE was generally higher during intermittent exercise compared to continuous exercise, despite similar intensities. Although there was no statistically significant difference in FS readings, SIT resulted in the lowest level of exercise experienced by individuals. The time-domain plots of the two metrics showed no significant differences between the intermittent exercise groups, but significant differences were observed with continuous exercise. The DMM results indicate that individuals remain in the process of moving from vigor-pleasure to fatigue-loss during both HIIT and THR. However, during SIT, individuals cross over to the vigor-loss quadrant in the final stage. Therefore, hypothesis (III) cannot be accepted. These results validate previous studies. Stork conducted a comparison between the correlation of exercise form and exercise experience. The results showed that longer periods of time (>30 min) of THR triggered smaller increases in RPE and decreases in FS in SB undergraduates compared to short (20 min) HIIT (Stork et al., 2018). In contrast, Yusuf found that HIIT resulted in higher levels of RPE and PA enjoyment scale (PACES) when comparing the physical and mental responses to the two forms of exercise (Soylu et al., 2021). The study suggests that population differences may be the cause of this phenomenon. While both this study and Yusuf’s study included college students, the inclusion criteria differed. Yusuf’s study included recreationally active students, while the present study included sedentary college students with low subjective interest in exercise. The intensity-varying form of exercise requires a high level of physical fitness, which SB students may lack. In terms of interval exercise, Nicole compared three different volumes of HIIT and found that a smaller volume resulted in higher levels of fatigue and a lower experience. This is consistent with the results of this study (Olney et al., 2018).

Two psychological models can explain the phenomenon and psychological mechanisms. According to Noakes’ central governor model (CGM), changes in muscle work during exercise are not determined by the metabolic factors of the muscle itself, but rather by the central nervous system (CNS). The CNS continuously integrates information from central, peripheral, and intracranial sensory afferents and actively adjusts the activity commands of motor units (alpha motor neurons and the skeletal muscle fibers they innervate) to achieve a constant load (Pompeu, 2022). During the process, THR experiences a constant load and therefore has a stable metabolic demand. On the other hand, SIT has a smaller volume and a more intensive transition between sprint and interval states, which places a greater demand on the CNS in terms of both muscular and metabolic activity. As a result, the supervisor experiences a decrease in fatigue and enjoyment. Marsden’s muscle wisdom hypothesis, on the other hand, suggests that CNS adaptation to changes in peripheral myocyte contractile behavior leads to a decrease in motor unit discharge frequency during muscle fatigue (Garland and Gossen, 2002). In the present study, the same frequent changes in muscle work efficiency forced by SIT resulted in constant adaptation of the CNS, which led to the onset of exercise fatigue more easily. Therefore, the present study concluded that HIIT and SIT are not suitable for the development of exercise interest among SB university students.

The study’s third finding revealed subtle differences in the physiological and psychological effects of training volume based on exercise loading. The results showed that, among the three sports, although there was no statistically significant difference between physiological load based on TRIMP and perceptual load based on s-RPE, THR had the lowest level of perceptual load and the highest physiological load based on the readings. Based on Edward’s TRIMP load intervals, it is evident that HIIT has the highest percentage of being in the high load zone (Zone-5), while THR has the smallest percentage and the smallest interval span. Therefore, part of hypothesis (IV) can be accepted. This result also explains the superiority of HIIT over the other two in terms of physiological benefits. However, the lowest perceptual load of THR also suggests that it produces less psychological stress on the individual. An effective exercise prescription minimizes perceptual loads and increases physiological loads. Therefore, many studies have compared subjective and physiological loads. Tannath monitored the ITL of rugby players at different stages of the off-season. The study found that increasing the intensity of training caused both loads to rise, but that stabilized form training reduced the individual’s s-RPE and perceptual load levels (Scott et al., 2022). Similarly, Jessica assessed load changes in ice hockey players over the course of a full season and found that the individual’s TRIMP expression levels were not greater than on weekdays, but s-RPE was significantly higher than on training days (Bigg et al., 2022). Therefore, it is suggested that changes in both the type of exercise and the environment can lead to increased perceptual loads, which may negatively impact an individual’s athletic performance. Additionally, Jill found that individuals had more consistent levels of fatigue expression during moderate to high intensity sustained exercise. However, TRIMP appeared to be higher in the HIIT group (Borresen and Lambert, 2008). The present study innovatively compared ITL between VPAs and found that training volume that is too intensive may not result in an increase in physiological load *versus* CRF benefits. The design of the training intervals affects the expression of perceptual load. Therefore, the study concludes that THR is more suitable for SB college students to acclimate during the initial period of exposure to sports. SIT and HIIT are suitable for the later period as an effective means to further improve the level of sports.

Based on these findings, previous evidence suggests that excessive training duration in SB adolescents inevitably leads to decreased exercise adherence. Therefore, studies have continued to examine the temporal characteristics of different exercise programs (Olney et al., 2018). In the present study, the duration of the three VPAs was approximately 20min per session, which is consistent with the goal of reducing training time to improve exercise efficiency. However, HIIT, SIT and THR still resulted in individual differences in physiological and perceived exertion due to differences in training volume. Based on the “Principle of Appropriate Load (Impellizzeri et al., 2022)”, this study suggests that SB adolescents can determine their own maximum intensity thresholds based on RPE at the beginning of their exercise exposure (Coe and Astorino, 2024). Due to the weak training base and poor exercise motivation of this group, they may not be able to ensure the integrity of their training during over-intensity HIIT and SIT. THR may be an effective way to increase interest in exercise and improve exercise adherence. Second, according to the “progressive overload training principle (Khushhal et al., 2020)”, the present study suggests that HIIT can be gradually incorporated into the training program and the intensity of the exercise can be increased after the exercisers have reached a certain level of baseline or exercise tolerance, for example, after one training cycle (2 months). Intensity to further promote the improvement of CRF and physiological adaptations. Finally, after a minimum of one and a half or volume cycles (3–4 months), SIT is used as an adjunct program to cross schedule with HIIT to enrich the workout, increase interest in the workout, and prevent the onset of central fatigue (Zarzissi et al., 2024).

## Limitations and suggestions for future research

This study aimed to compare the acute effects produced by three groups of VPA of similar intensity on CRF indexes of sedentary college students by means of load monitoring, and to assess the differences between their subjective and objective reflections. The resolution and fine control of exercise were taken as the core of exploration. The goal was to determine the optimal exercise dose to enhance college students’ CRF and cultivate their interest in exercise. However, this study still has limitations and deficiencies in terms of experimental design and training control.

The main objective of this study was to investigate whether differences in training volume during exercise of similar intensity affect the cardiac beat characteristics, respiratory metabolic status, and exercise experience of individuals. Additionally, the study aimed to assess the long-term post-exercise effects through acute effects. Strictly speaking, the experimental design of the present study, using cross-over repeated measures, is more reasonable. This approach better demonstrates the rigor of the experiment through the use of a homologous control. The initial experimental design of this study also adopted this scheme. However, the physical condition of the subjects and the arrangement of the actual intervention were not well controlled. During the homozygous controls, it was observed that the majority of subjects had less than 30% success and adherence to training after one to two single interventions (with a recovery interval of at least 72 h). As a result, only four out of the initial 10 completed the 3-sport intervention within 4 weeks, and the heterogeneity of results was too great to be included in the normal results. Therefore, the present study remedied this problem through a randomized controlled approach. Although this design requires a high number of samples, the present study included only 10 male participants in each group to minimize experimental error, as required.

The study focused on the acute effect of three exercise conditions on CRF metrics and subjective exercise experience in sedentary college students. The results indicate which form of exercise has the best overall improvement effect on individuals after a single session of VPA. If exploring the long-term effects of PA, the present study should be adapted to a longitudinal study with a long-term intervention for individuals. However, the experimental samples included in this study were comprised solely of undergraduate and graduate students with sedentary habits. As a result, this population had a low baseline level of exercise and interest in independent exercise, as well as class scheduling issues, which made it more difficult to organize long-term interventions. Additionally, adherence to homogeneous implementation was extremely low. However, this study is still in the process of testing the intervention in stages, with a limited number of subjects, 2–3 times a week. During successive interventions, it was found that due to the implementation of VPA, which requires a high level of physical fitness, the heterogeneity of the indicators during each monitoring was greater compared to MICT. This is why most studies have used MICT with HIIT as a control. However, this study focuses on the control of HIIT, SIT, and THR. Therefore, after careful consideration, we finalized the study design as a cross-sectional control of acute exercise.

Therefore, in the future, this study will focus on improving and optimizing the experimental design. This will involve implementing repeated cross-measurements under limited conditions to monitor individuals more precisely in different PAs. To improve the persuasiveness and rigor of the experimental results, this study will implement a longitudinal continuous intervention for existing subjects and strive to improve adherence by encouraging and adjusting the recovery interval. No new content has been added. This approach aims to comprehensively assess the optimal dose of PA to improve CRF and exercise interest in sedentary college students. It provides a more efficient and specific exercise prescription for non-pharmacological interventions to improve sedentary behavior.

## Conclusion

(1) Differences in training volume at similar intensities mediate varying levels of respiratory metabolism. HIIT triggers the highest levels of VO2, VCO2, and OP, making it the most effective single-session stimulus for CRF. (2) The type of PA has a direct impact on an individual’s PA experience. Intermittent PA can lead to excessive fatigue and reduce the perception of PA, which is not helpful in developing interest in PA among sedentary college students. (3) Differences in training volume at similar intensities cause variations in both subjective and physiological loads. Sustained exercise results in the lowest perceptual loads and the highest physiological loads, which are suitable for adapting to and establishing PA habits. (4) During the habit-establishment period, using a moderate amount of THR to cultivate interest and adaptive intensity, and the use of HIIT to enhance workout efficiency during the adaptation period, along with SIT to reduce the monotony of training, may be an effective way to improve the cardiorespiratory fitness of sedentary college students and to establish PA habits.

## Data Availability

The original contributions presented in the study are included in the article/Supplementary material, further inquiries can be directed to the corresponding authors.
